# Development and validation of epithelial mesenchymal transition-related prognostic model for hepatocellular carcinoma

**DOI:** 10.18632/aging.202976

**Published:** 2021-04-30

**Authors:** Xuequan Wang, Ziming Xing, Huihui Xu, Haihua Yang, Tongjing Xing

**Affiliations:** 1 Public Research Platform, Department of Radiation Oncology, Taizhou Hospital of Zhejiang Province affiliated to Wenzhou Medical University, Linhai 317000, Zhejiang Province, China; 2 School of Biological Science and Medical Engineering, Beihang University, Beijing 100191, China; 3 Central Laboratory, Taizhou Hospital of Zhejiang Province affiliated to Wenzhou Medical University, Linhai 317000, Zhejiang Province, China; 4 Department of Radiation Oncology, Taizhou Hospital of Zhejiang Province affiliated to Wenzhou Medical University, Linhai 317000, Zhejiang Province, China; 5 Department of Infectious Disease, Taizhou Hospital of Zhejiang Province affiliated to Wenzhou Medical University, Linhai 317000, Zhejiang Province, China

**Keywords:** hepatocellular carcinoma, epithelial cell transformation, prognosis

## Abstract

Epithelial cell transformation (EMT) plays an important role in the pathogenesis and metastasis of hepatocellular carcinoma (HCC). We aimed to establish a genetic risk model to evaluate HCC prognosis based on the expression levels of EMT-related genes. The data of HCC patients were collected from TCGA and ICGC databases. Gene expression differential analysis, univariate analysis, and lasso combined with stepwise Cox regression were used to construct the prognostic model. Kaplan–Meier curve, receiver operating characteristic (ROC) curve, calibration analysis, Harrell’s concordance index (C-index), and decision curve analysis (DCA) were used to evaluate the predictive ability of the risk model or nomogram. GO and KEGG were used to analyze differently expressed EMT genes, or genes that directly or indirectly interact with the risk-associated genes. A 10-gene signature, including *TSC2*, *ACTA2*, *SLC2A1*, *PGF*, *MYCN*, *PIK3R1*, *EOMES*, *BDNF*, *ZNF746*, and *TFDP3*, was identified. Kaplan–Meier survival analysis showed a significant prognostic difference between high- and low-risk groups of patients. ROC curve analysis showed that the risk score model could effectively predict the 1-, 3-, and 5-year overall survival rates of patients with HCC. The nomogram showed a stronger predictive effect than clinical indicators. C-index, DCA, and calibration analysis demonstrated that the risk score and nomogram had high accuracy. The single sample gene set enrichment analysis results confirmed significant differences in the types of infiltrating immune cells between patients in the high- and low-risk groups. This study established a new prediction model of risk gene signature for predicting prognosis in patients with HCC, and provides a new molecular tool for the clinical evaluation of HCC prognosis.

## INTRODUCTION

Hepatocellular carcinoma (HCC) is one of the most life-threatening tumors worldwide. According to current statistics, HCC is the sixth-leading cause of cancer and the fourth-leading cause of cancer death worldwide [1]. HCC is also the fourth most common malignant tumor and the second-leading cause of cancer death in China, with 500,000 new confirmed cases and more than 400,000 deaths each year in China [2, 3]. In recent decades, great progress has been made in the diagnosis and treatment of HCC. The 5-year survival rate has reached more than 50% after surgery for early-stage HCC, but the recurrence rate is still relatively high. The treatment methods for advanced HCC are limited, and the prognosis is poor. In recent years, molecular and immune-targeted therapies such as Lenvatinib, programmed cell death protein 1 (PD-1) monoclonal antibodies, and bevacizumab have achieved good results in HCC; however, they still face problems such as high cost, poor targeting, and adverse reactions [4, 5]. How to scientifically evaluate the prognosis of HCC patients and implement precise treatment is currently a hot topic in the field of HCC research.

HCC is highly heterogeneous, and commonly used indicators such as α-fetoprotein (AFP), D-γ-hydroxyprothrombin (DCP), and cytokeratin (CK)-18 are unable to meet the clinical prognosis evaluation needs of patients with HCC. Previous studies that have examined single or multiple genes have found several molecular markers for predicting HCC prognosis [6, 7]. The development of genomics, proteomics, and metabolomics has helped to establish the corresponding molecular typing of HCC and provide new means and indicators for its prognosis [8–10]. However, high-throughput sequencing and large-scale gene mutation detection have not yet solved the obstacles that challenge the precise diagnosis and treatment of HCC, including the variety of HCC types and the technical difficulty in identifying them. To address this problem, several scholars have recently established small panel gene prediction models to predict the prognosis of HCC [11–13], which have shown better predictive accuracy than single biomarkers. The application of epithelial mesenchymal transition (EMT)-related genes in predicting HCC prognosis has not yet been reported.

The poor prognosis of HCC occurs primarily because HCC is prone to metastasis. There are many factors related to the mechanisms of tumorigenesis and metastasis, and EMT and its reverse process are important underlying mechanisms [14]. After EMT occurs, cell adhesion decreases and the movement and invasion capacity increases; this allows tumor cells to detach from the primary lesion and enter the peripheral blood vessels and lymphatic system [15]. The reverse process of EMT—mesenchymal epithelial cell transformation (MET)—is conducive to the homing and proliferation of tumor cells to form metastases. EMT involves multiple signaling pathways and complex molecular mechanisms, including the TGF-beta, WNT, and FGF signaling pathways [16]. A database of EMT-related genes has been established by some scholars [17]. Considering the above, we examined EMT-related genes and applied the HCC data to The Cancer Genome Atlas (TCGA) for bioinformatics analysis. We then screened the EMT genes that are closely related to HCC prognosis to establish a molecular prediction model to evaluate HCC prognosis. We validated the prediction model in an International Cancer Genome Consortium (ICGC) data cohort. This provided a new molecular model to evaluate the prognosis of patients with HCC and guide clinical diagnosis and treatment.

## MATERIALS AND METHODS

### Data acquisition and arrangement

The mRNA expression RNA-Seq data and DNA methylation data were analyzed by an Illumina Human Methylation 450 BeadChip, and clinical information about liver hepatocellular carcinoma (LIHC) was acquired from the TCGA (https://cancergenome.nih.gov/)-LIHC database to analyze differentially expressed genes and build gene prognostic models. ICGC data were downloaded from the ICGC database (https://icgc.org/) for external validation of the prognostic gene models. EMT-related genes were collected from the dbEMT database [17].

### Identification of differentially expressed genes in HCC

The raw RNA-seq data were annotated and then normalized by the variance stabilizing transformation (VST) function [18]. The differential gene expressions of the TCGA-LIHC tumor samples were analyzed against normal samples. The absolute value thresholds of the log2 fold changes (logFC) > 0.5 and adjusted *P* value < 0.0001 were adopted for EMT-related genes. A volcanic map and heat map were constructed to illustrate the EMT genes that were differentially expressed between cancer tissues and normal tissues.

### Establishment of a prognostic gene signature and internal verification

Univariate Cox regression analysis was performed to screen the EMT genes related to prognosis. A *P*-value < 0.05 was considered to indicate statistical significance. Next, the least absolute shrinkage and selection operator (lasso) regression model was performed to minimize over-fitting and identify the most significant survival-associated differentially expressed EMT-related genes in HCC. Stepwise multivariate Cox regression analysis was performed after testing for collinearity to establish the EMT-derived risk signature in HCC. The risk score was calculated by the following formula based on a combination of the Cox coefficient and gene expression [19]:

$$Risk\;score\;=\;\sum_{i\;=\;1}^{K}\beta \;i*\;Expi,$$

where k, βi, and Expi represent the number of signature genes, the coefficient index, and the gene expression level, respectively.

To verify the single-gene prognostic value of the model’s corresponding EMT-related genes, patients were divided into high- and low-expression groups based on the gene expression level as determined by the surv_cutpoint function in the “survminer” package. The time-dependent prognostic value of the gene signature, together with pathologic TNM staging, age, tumor grade or tumor stage was investigated by the Kaplan–Meier (KM) method, and the log-rank test was used to compare the survival difference between the high- and low-expression groups. The patients were sorted by risk score, and the risk score distribution and survival status for each patient, as well as the heat map of the risk-associated gene expression levels were determined.

To detect the significance of EMT in HCC diagnosis, the independent subsets were randomly categorized into a training set and an internal validation set using the “caret” package. Receiver operating characteristic (ROC) analysis was performed with independent subsets of the TCGA-LIHC samples, and the area under the ROC curve (AUC) was calculated. The predictive value of the prognostic gene signature was further studied in the test cohort and later verified in the ICGC Japanese HCC cohort.

### Protein expression and gene mutation analysis of the prognostic gene signature

The protein expression levels of the prognosis-related genes in normal tissue and HCC tissue were retrieved from the Human Protein Atlas database, and the data were visualized in immunohistochemistry staining images (http://www.proteinatlas.org). The mutation types of the risk-related genes were explored using the “TCGAmutations” package.

### Independent prognostic role of the gene signature in TCGA

The risk score of each HCC sample and the corresponding clinical factors (including age, sex, tumor grade, tumor stage, and pathologic TNM staging) were subjected to univariate and multivariate Cox regression analyses. Univariate and multivariate Cox regression analysis were performed to investigate whether the prognostic gene signature or the individual genes could be independent prognostic indicators after combing the clinical parameters. *P*-values < 0.05 indicated statistical significance.

### Identification of candidate methylation prognostic indicators from the signature-related genes

Methylation sites in the signature-related genes were collected from the methylation annotation file. Overall, 379 tumor samples and 50 normal samples with 340 DNA methylation sites were analyzed. The methylation value of each methylation site in liver cancer tissues and normal tissues is displayed in a scatter diagram according to the arrangement of methylation sites. The methylation data of tumor tissue matched with patient full survival information were used to assess the association between DNA methylation levels and the overall survival (OS) by univariate Cox regression analysis. Besides, the DNA methylation status of signature-related genes and their expression levels, clinical information, and survival status were also investigated using MEX.PRESS (http://mexpress.be).

### Building and validating a predictive nomogram

By employing “survival,” “foreign,” and “rms” packages, a nomogram consisting of relevant clinical parameters and independent prognostic factors, based on previous independent prognostic factor screening, was used to predict the probability of 1-, 3-, and 5-year OS in patients with HCC. The nomogram performance was evaluated by Harrell’s concordance index (C-index) [20]. A time-dependent ROC curve and the calibration curve were also plotted to estimate the accuracy of the observed rates compared to the nomogram’s predicted survival for the 1-, 3-, and 5-year OS categories. Moreover, the clinical application prospects of the 10-gene signature were determined by decision curve analysis (DCA) [21].

### Identification of the relationship between the risk score and the immune landscape

A single simple gene set enrichment analysis (ssGSEA) method was performed using the “GSVA” package to clarify the relationship between the risk score and the immune landscape. The enrichment analysis depended on the gene sets for 28 types of specifically labeled tumor immune-infiltrating cells (TIICs) [22]. Corresponding scores to reflect the infiltration abundance of each single TIIC sample were collected through the enrichment analysis, and the enrichment scores of the high- and low-risk groups were analyzed. Additionally, the correlation was calculated between the risk score and the immune landscape using the “ggstatsplot” package (https://github.com/Indrajeet.Patil/ggstatsplot/issues). The cut-off of the *P*-values obtained by an independent samples t-test was *P* < 0.05.

### Protein-protein interaction network

We next sought to evaluate the functional enrichment of the ten risk-related genes and to better understand the efficacy of the risk model. To this end, we collected the proteins that had been confirmed to interact directly or indirectly with the ten risk genes obtained using the Search Tool for the Retrieval of Interacting Genes (STRING) database [23]. The protein interaction networks were constructed with a confidence > 0.4 as the cut-off.

### Functional enrichment analysis

Functional enrichment analysis was performed using the Kyoto Encyclopedia of Genes and Genomes (KEGG) and Gene Ontology (GO) analyses to explore the biological function of the HCC-specific EMT genes or the protein-protein interaction network-related genes. The “clusterProfiler” package in R software was used to determine the cellular component, the molecular function, and the biological process for GO analysis, and pathway analysis in KEGG [24]. The histograms for the results of functional enrichment analysis were acquired by the “GO plot” R package. An adjusted *P* < 0.001 was used as the threshold.

### Statistical analysis

R software 3.6.3 (http://www.Rproject.org) was used for statistical analyses (Vienna, Austria). The “Deseq2 R” package was used to nominalize the data, identify differentially expressed genes, and to perform principal component analysis. The “ggplot2” was used to plot the heat map, volcano plot, boxplot, and scatter plot. The “My.stepwise,” “survival,” and “survminer” packages were used for Cox regression and Kaplan–Meier survival analyses. Lasso regression, ROC curve, DCA, and C-index were performed in “glmnet,” ”timeROC,” “rmda,” and “survcomp” packages. Nomogram and corresponding calibration curves were performed using the “rms” package. All of the other packages used are mentioned above. Qualitative variables were analyzed by χ^2^ test or Fisher’s exact test. Quantitative variables were analyzed by t-test or nonparametric Wilcoxon rank-sum test.

## RESULTS

### Identification of HCC-specific EMT genes and their functional enrichment

**Figure 1:**
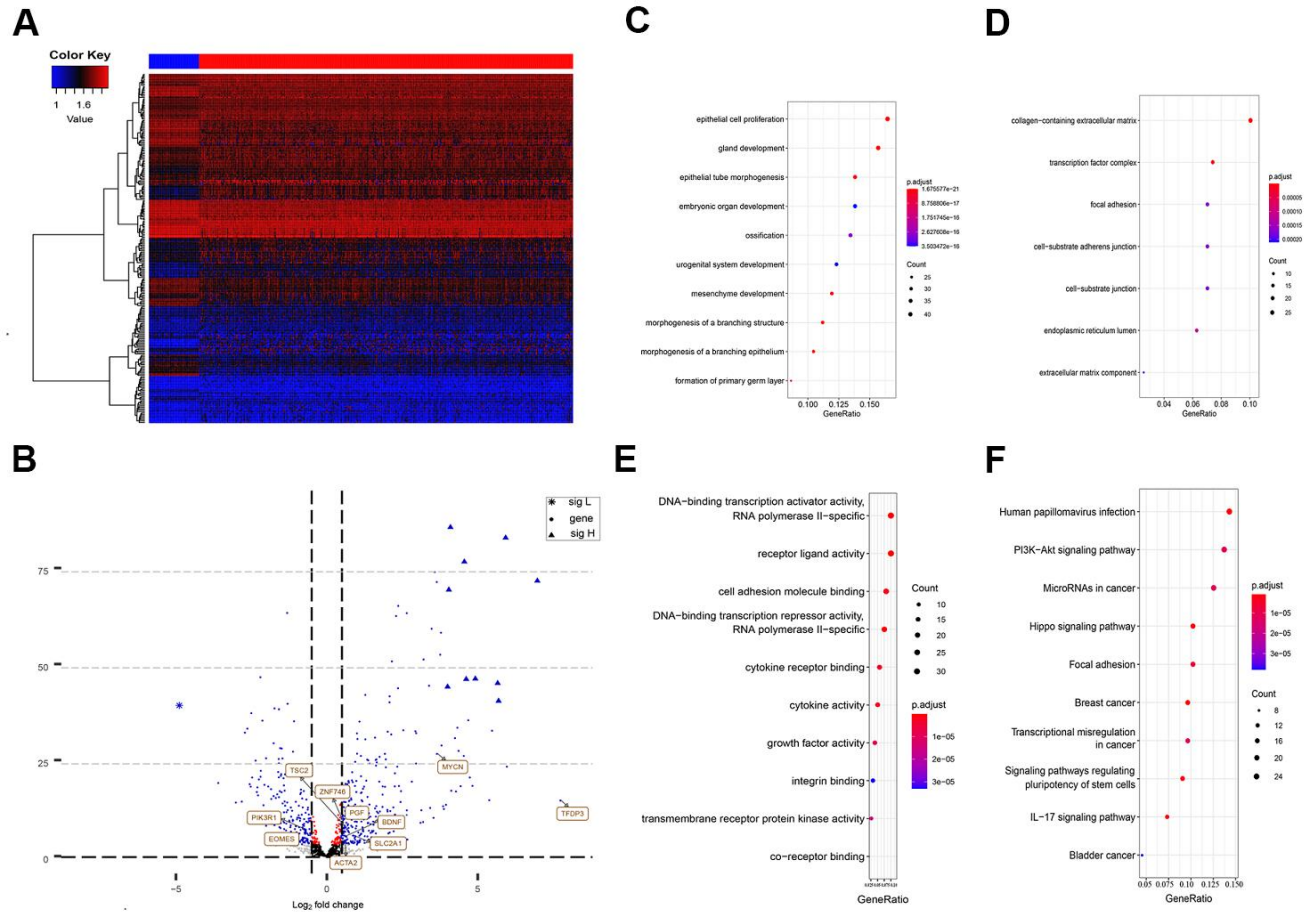
**Differently expressed EMT related genes between normal and HCC tissues.** (**A**) Heatmap of 270 differently expressed EMT related genes in TCGA-LIHC. The color from blue to red represents low expression to high expression. (**B**) Volcano plot of EMT related genes: blue indicates down and upregulated genes. Changes of the risk genes are identified by names. (**C**–**E**) Dot plots represents biological process, cellular component and molecular function of Gene ontology analysis based on 270 HCC dysregulated EMT related genes respectively. (**F**) KEGG pathway analysis of differently expressed EMT related genes. EMT, Epithelial cell transformation; HCC, hepatocellular carcinoma; TCGA, The Cancer Genome Atlas; KEGG, Kyoto Encyclopedia of Genes and Genomes.

The differential analysis of the sequencing data, including 50 normal samples and 374 tumor samples, showed that 2,547 genes among the total 47,781 annotated genes were significantly abnormally expressed (with a cut-off of absolute log2FC > 2 and *P* < 0.0001). These dysregulated genes included 64 EMT-related genes, which were significantly differentially expressed in HCC tissues and normal tissues. A heat map for the expression profile of the 270 differently expressed EMT-related genes with absolute log2FC > 1 and *P* < 0.0001 cut-offs are shown in Figure 1A. Abnormally expressed changes in EMT-related genes are displayed in the volcano plot, and the majority of genes were upregulated (Figure 1B).

According to the functional enrichment analysis, the 270 EMT genes were mainly enriched in 634 GO terms and 68 KEGG pathways (*P* < 0.001). These results indicated that most of the EMT genes participated in biological processes. The top molecular functions of these 270 HCC-specific EMT genes included protein, RNA, and DNA binding, and enzyme activity, such as DNA-binding transcription activator activity, RNA polymerase II-specific co-receptor binding, and transmembrane receptor protein kinase activity (Figure 1E). In terms of biological processes, the majority of these genes were involved in epithelial cell proliferation (Figure 1C). These genes are the main cell component of the collagen-containing extracellular matrix (Figure 1D).

The KEGG analysis suggested that these HCC-specific EMT genes were mainly involved in pathways associated with cancer, phosphatidylinositol-3-kinase (PI3K)/Akt signaling, focal adhesion, regulation of stem cell pluripotency, IL-17 signaling, transcriptional misregulation in cancer, Wnt signaling, and TNF signaling (Figure 1F).

### Establishment and internal validation of the ten prognostic genes in TCGA-LIHC

Excluding samples with incomplete OS data or clinical stages, 346 TCGA-LIHC patients with a follow-up time of more than 30 days were included for subsequent analysis. In total, 106 genes associated with HCC OS in the TCGA-LIHC cohort were screened using univariate Cox regression analysis and different expression results.

**Figure 2:**
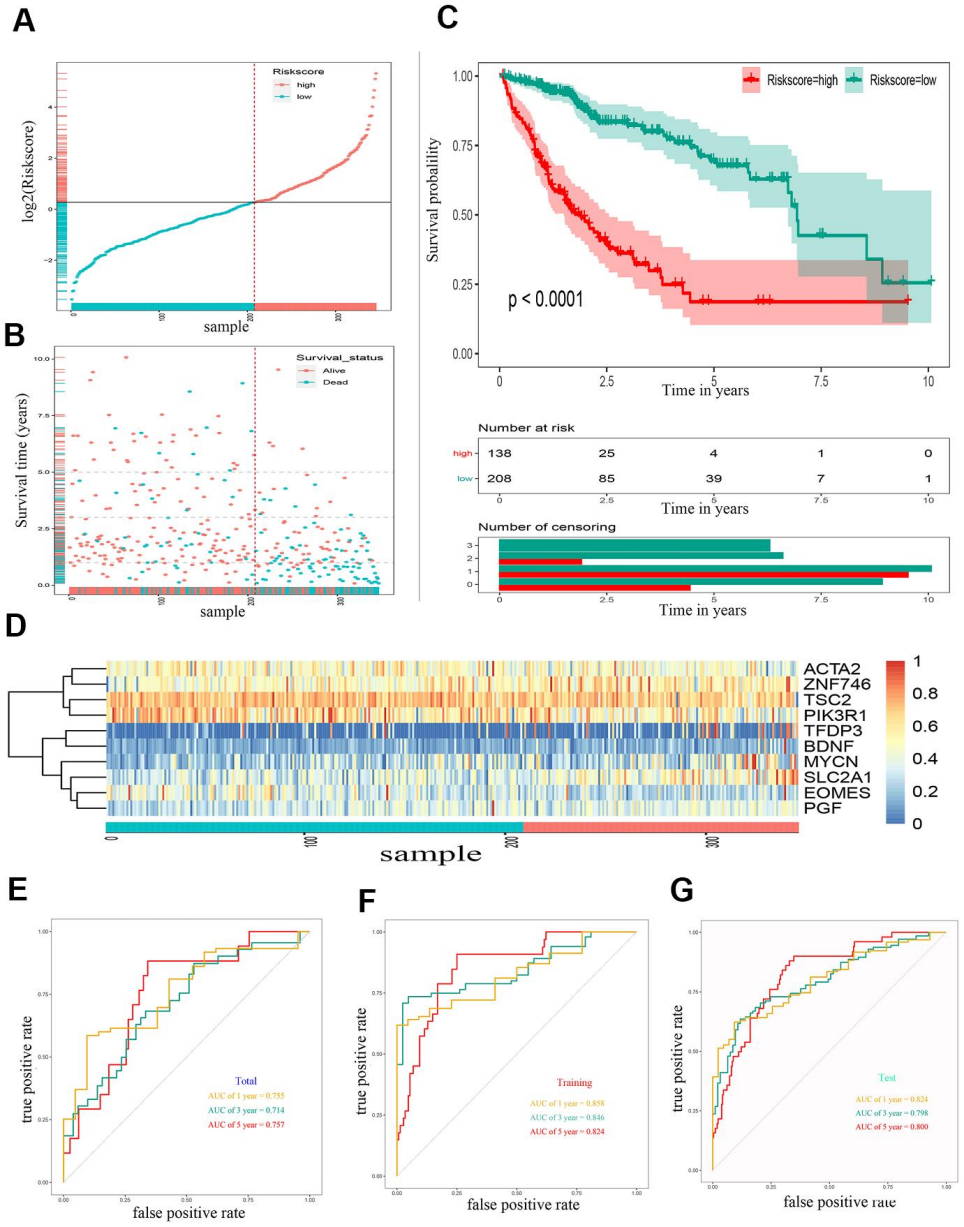
**Construction of a 10 gene based EMT-related prognostic signature for HCC in TCGA-LIHC cohort.** (**A**) The risk score distribution of HCC patients in TCGA-LIHC cohorts. (**B**) Duration and survival status of HCC patients. (**C**) Kaplan-Meier analysis of ten-gene signature in TCGA-LIHC cohort. (**D**) Heatmaps of the ten gene signature relative expression. (**E**) Time-dependent ROC analysis of ten-gene signature in training set. (**F**) Time-dependent ROC analysis of ten-gene signature in testing set. (**G**) Time-dependent ROC analysis of ten-gene signature in total cohort. HCC, hepatocellular carcinoma; TCGA, The Cancer Genome Atlas; ROC, receiver operating characteristic curve.

Through lasso Cox regression, we further narrowed the number of genes associated with OS to 22 (Supplementary Figure 1). Subsequently, an optimal prognostic signature based on ten EMT-related genes was identified by stepwise Cox analysis. The identified genes included tuberous sclerosis 2 (*TSC2*), actin alpha 2 (*ACTA2*), solute carrier family 2 member 1 (*SLC2A1*), placental growth factor (*PGF*), mycn proto-oncogene (*MYCN*), phosphoinositide-3-kinase regulatory subunit 1 (*PIK3R1*), eomesodermin (*EOMES*), brain-derived neurotrophic factor (*BDNF*), zinc finger protein 746 (*ZNF746*), and transcription factor Dp family member 3 (*TFDP3*). The expression of all ten risk genes in HCC was dysregulated (absolute logFC > 0.5, *P* < 0.0001), and only *PIK3R1* and *EOMES* were downregulated. The relative gene expression levels of the ten prognostic genes after the sample ordered by risk score are shown in the heat map (Figure 2D). The patients were then stratified into low- and high-risk groups at the best separation cut-off of the risk score, and the risk score distribution of each patient is shown in Figure 2A. The survival of patients in the two groups was significantly different. The mean survival time of the high-risk patients was significantly shorter, and the vital status of death accounted for a larger proportion than that of low-risk patients (Figure 2B). Kaplan–Meier survival curve analysis revealed that the prognoses of patients in the high-risk group were significantly worse than those of patients in the low-risk group (Figure 2C). Kaplan–Meier survival curve analysis also showed that the expression of the ten genes, except *BDNF*, could distinguish the survival probability of patients with HCC (Supplementary Figure 2). A total of 346 patients were randomly divided into a training group (n = 200 cases) and a validation group (n = 146 cases). The AUCs for 1-, 3, and 5-year OS in the training group were 0.858, 0.846, and 0.824, respectively (Figure 2E). The AUCs for 1-, 3-, and 5-year OS in the validation group were 0.755, 0.714, and 0.757, respectively (Figure 2F). The AUCs for 1-, 3-, and 5-year OS in the overall sample were 0.824, 0.798, and 0.800, respectively (Figure 2G).

### External validation of the gene signature in the ICGC cohort

**Figure 3:**
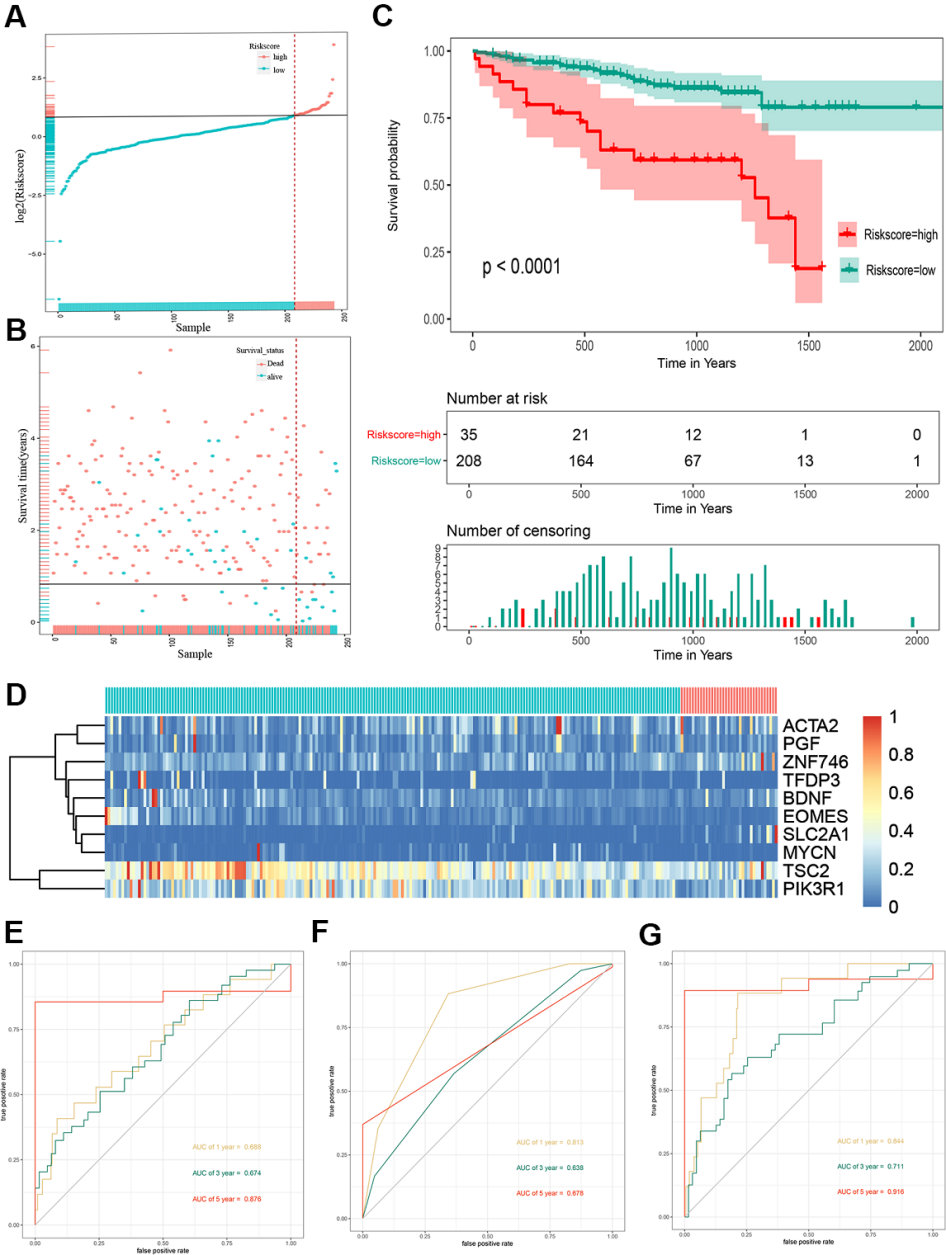
**Risk score analysis, Kaplan-Meier analysis and time-dependent ROC analysis of ten-gene signature in ICGC cohort.** (**A**) Risk score analysis of ten-gene signature in ICGC cohort. (**B**) Distribution of risk score. (**C**) Kaplan-Meier analysis of ten-gene signature in ICGC cohort. (**D**) Heatmap of ten gene signature. (**E**) Time-dependent ROC analysis of ten-gene signature in total cohort. (**F**) Time-dependent ROC analysis of ten-gene signature in training set. (**G**) Time-dependent ROC analysis of ten-gene signature in testing set. ROC, receiver operating characteristic curve; ICGC, International Cancer Genome Consortium.

To validate the outcome model based on the ten EMT-related genes, 243 Japanese patients with HCC from the ICGC cohort were used for the external validation. The patients were divided into high- and low-risk groups by the optimal risk score cut-off, and the low-risk group had a better prognosis than the high-risk group (Figure 3A). The OS of high-risk patients was significantly worse than that of low-risk patients (Figure 3B). Kaplan–Meier survival curve analysis showed a significant difference in the OS between the two groups (*P* < 0.0001) (Figure 3C). A heat map of the 10-gene signature is shown in Figure 3D. The AUCs for 1-, 3-, and 5-year OS predictions for risk score were 0.688, 0.674, and 0.876, respectively (Figure 3E). The AUCs for 1-, 3-, and 5-year OS predictions for based on tumor stage were 0.813, 0.638, and 0.678, respectively (Figure 3F). The AUCs improved when combined with the stage, age, and 10-gene signature, with values of 0.844, 0.711, and 0.916 for 1-, 3-, and 5-year OS, respectively (Figure 3G).

### Building and validating a predictive nomogram

**Figure 4:**
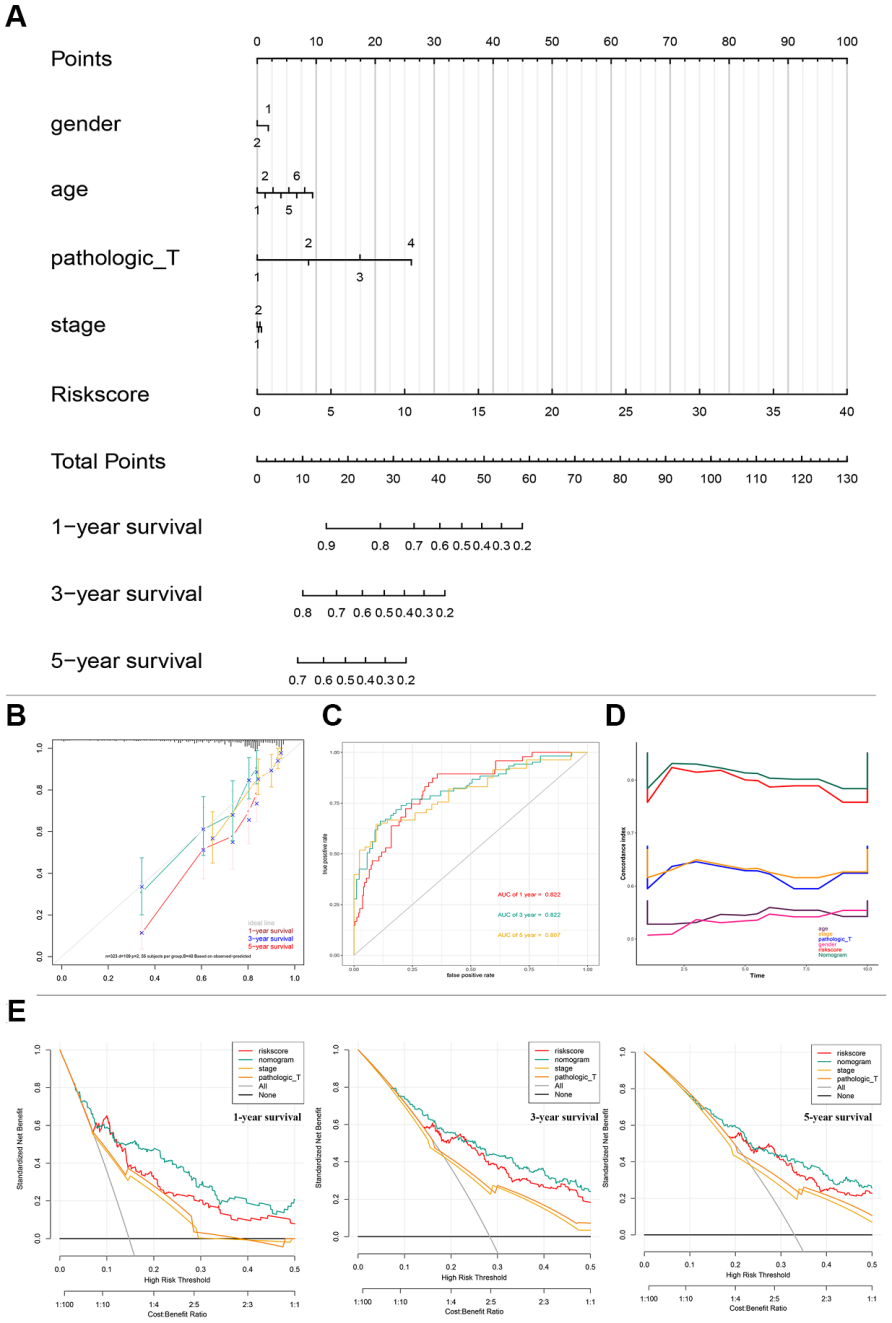
**Nomogram predicting overall survival for HCC patients in TCGA cohorts.** (**A**) A prognostic nomogram predicting 1-, 3-, and 5- year OS of HCC. (**B**) Calibration plots of the nomogram. (**C**) Time-dependent ROC analysis of nomogram predicting 1-, 3-, and 5- year OS of HCC. (**D**) C-index of the nomogram. (**E**) Decision curve analysis of nomogram predicting 1-, 3-, and 5- year OS of HCC comparing the risk score, stage and Pathologic T. HCC, hepatocellular carcinoma; TCGA, The Cancer Genome Atlas; OS, overall survival; ROC, receiver operating characteristic curve.

To better predict the prognosis of patients with HCC, a prognostic nomogram was constructed based on 323 HCC patients with complete clinical information about risk score combing clinical factors (age, sex, stage, pathologic T) (Figure 4A). The AUCs of 1-, 3-, and 5-year OS predictions were 0.822, 0.822, and 0.807, respectively (Figure 4C). The calibration plots showed that the nomogram was best for predicting 1-, 3-, and 5-year OS in patients with HCC (Figure 4B). The C-index values of the nomogram were 0.812, 0.799, and 0.633 respectively for 1-, 3-, and 5-year OS (*P* < 0.0001), suggesting that the performance of the nomogram was reliable (Figure 4D). DCA showed the nomogram performed better at the threshold probability (ranging from 2% to 50%) in Figure 4E. These results revealed that the prognostic nomogram was superior in predicting the 1-, 3-, and 5-year survival outcomes of patients with HCC.

### Comparison of predictive ability between risk score and clinical indicators

**Figure 5:**
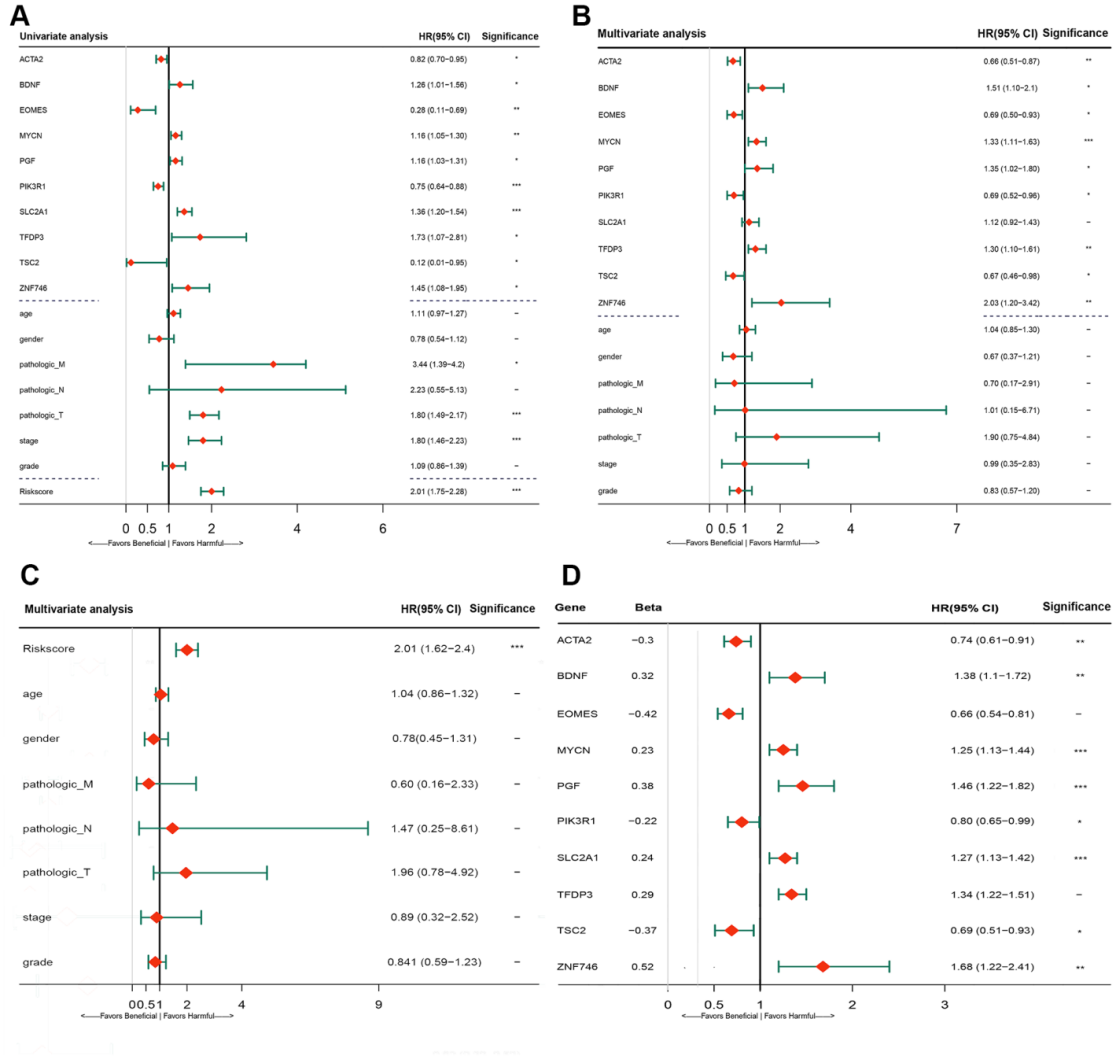
**Forrest plot of the univariate and multivariate Cox regression analysis in TCGA cohorts.** (**A**) Forrest plot of the univariate Cox regression analysis OS of 10 gene signature and clinical factor. (**B**) Forrest plot of the multivariate Cox regression analysis OS of 10 gene signature and clinical factor. (**C**) Forrest plot of the multivariate Cox regression analysis OS of risk score and clinical factor. (**D**) Forrest plot of the multivariate Cox regression analysis OS of 10 gene signature. Beta values representatives the coefficient index β for each gene. TCGA, The Cancer Genome Atlas; OS, overall survival.

**Figure 6:**
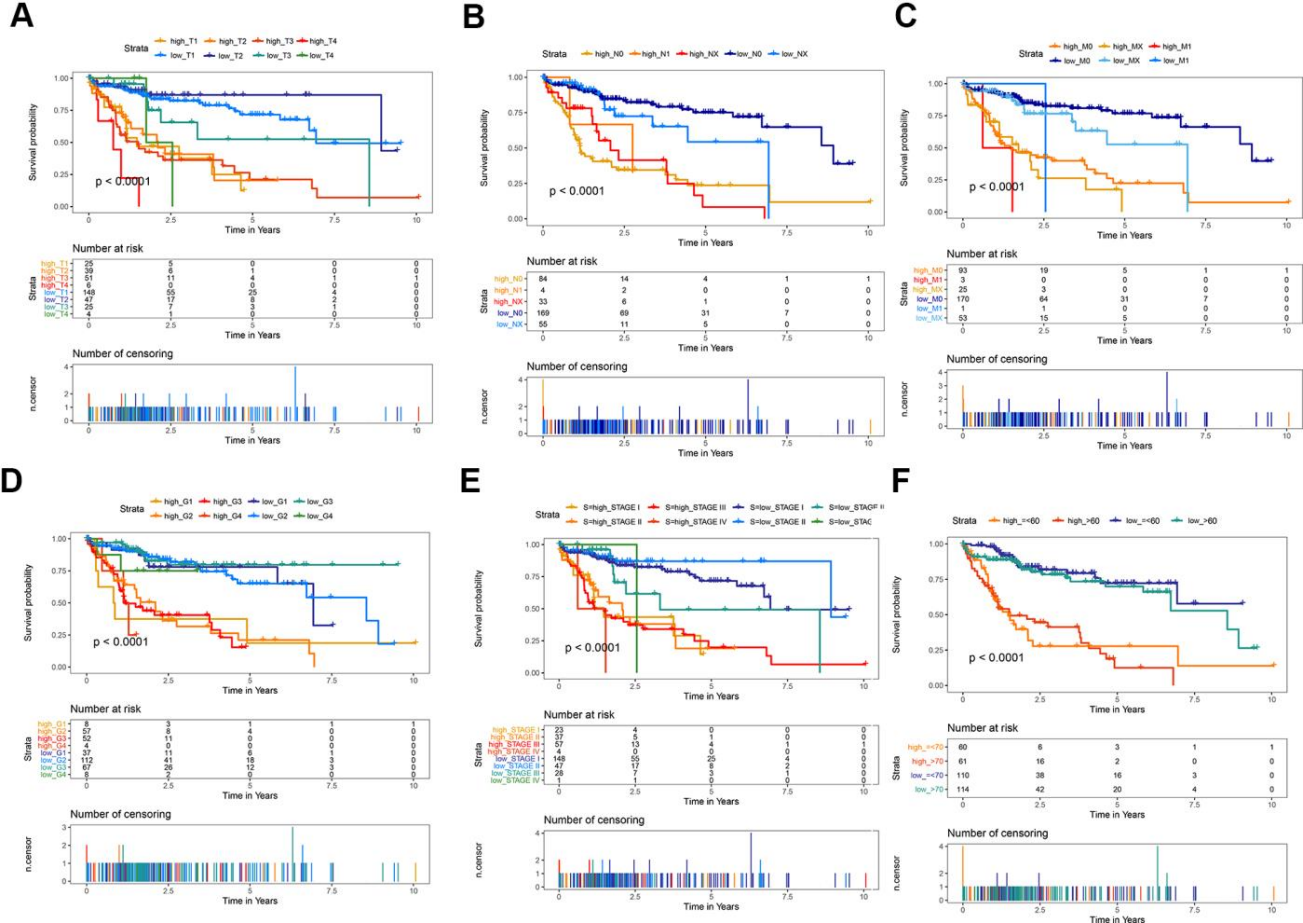
**Kaplan-Meier survival analysis of the gene signature compared with clinical indicators.** (**A**) T stage; (**B**) N stage; (**C**) M stage; (**D**) tumor grade; (**E**) AJCC stage; (**F**) Age.

The predictive ability of the risk score was compared with the following clinical indicators: age, sex, pathologic TNM staging, and HCC stage. Univariate Cox regression analysis showed that risk score, stage, and pathologic T and M stages were risk factors for poor prognosis (Figure 5A). Multivariate Cox regression analysis showed that only the risk score was an independent risk factor for poor prognosis of HCC (Figure 5B–5D). Both univariate and multivariate Cox regression analyses were statistically significant. These results suggested that the ten EMT-related gene-based signature was an independent prognostic indicator for HCC OS. Kaplan–Meier survival analysis showed that the gene signature could better predict the prognosis of patients with HCC compared with the commonly used TNM classification, AJCC stage, tumor grade, and age (Figure 6). The AUC of the risk score was significantly higher than those of other clinical factors for predicting 1-, 3-, and 5-year OS in patients with HCC (Table 1). The risk score could not only distinguish between normal and tumor tissues, but could also show differences in tumor grade and HCC stage (Supplementary Figure 3).

**Table 1:** Comparison of predictive ability of risk score and clinical indicator.

**OS**	**Age**	**Sex**	**TNM**	**Pathologic T**	**Grade**	**Risk score**
1 year OS	0.509	0.508	0.712	0.721	0.494	0.833
3 year OS	0.497	0.520	0.681	0.681	0.518	0.820
5 year OS	0.573	0.516	0.645	0.662	0.558	0.814

### Genetic variation and protein expression of the 10-gene signature

The protein expression of the ten genes was explored in the Human Protein Profiles. The characteristic picture of seven genes is shown in Supplementary Figure 4C. *BDNF* had lower expression in hepatocytes and was not detected in tumor cells. *PIK3R1* had lower expression in hepatocytes and medium expression in tumor cells. The others genes showed similar expression between hepatocytes and tumor cells. The protein expression of *PGF*, *MYCN*, and *TFDP3* were not found in this database.

Gene mutation analysis showed that 26 of the 363 samples (7.16%) had mutations in six genes (Supplementary Figure 4A). Among these genes, the *TSC2* gene is most susceptible to mutation, and its mutations include missense mutations, nonsense mutations, and frame shifts (Supplementary Figure 4B). Missense mutation was also the most common genetic alteration for *PIK3R1*, *EOMES*, *MYCN*, *ZNF746*, and *BNDF*. Owing to the limitation of the sample of mutations, no relevant survival analysis related to each mutated gene was performed.

### Prognostic-related gene methylation status of the 10-gene signature

**Figure 7:**
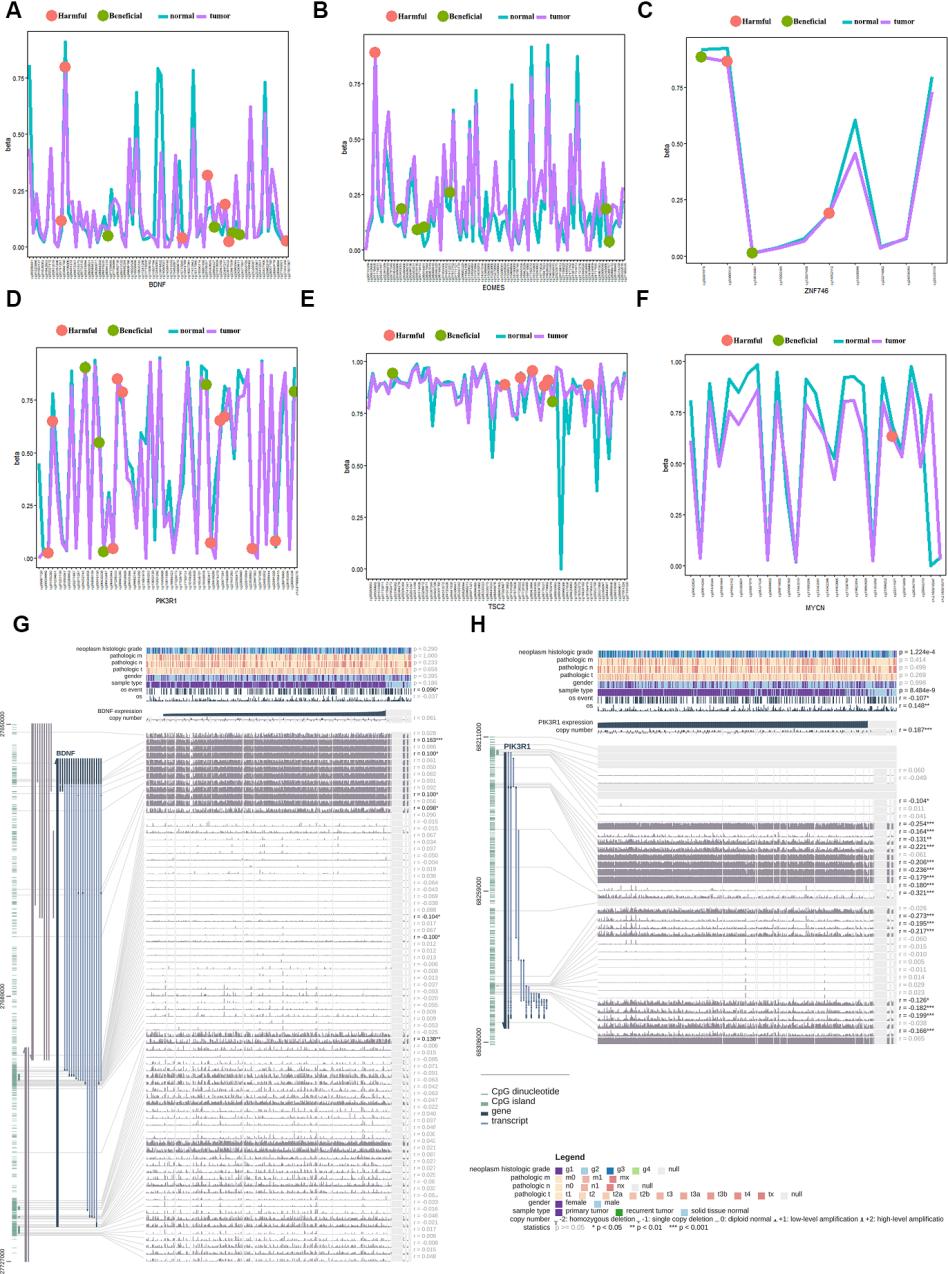
**Gene methylation analysis of the gene signature between the tumor and normal tissues.** (**A**) beneficial and harmful methylation sites in BDNF; (**B**) beneficial and harmful methylation sites in EOMES; (**C**) beneficial and harmful methylation sites in ZNF746 gene; (**D**) beneficial and harmful methylation sites in PIK3R1gene; (**E**) beneficial and harmful methylation sites in TSC2 gene; (**F**) beneficial and harmful methylation sites in MYCN gene; (**G**) The methylation of BDNF gene significantly correlated with the OS of HCC patients; (**H**) The methylation of PIK3R1 gene significantly correlated with the OS of HCC patients. HCC, hepatocellular carcinoma; OS, overall survival; ROC, receiver operating characteristic curve.

Univariate Cox regression analysis demonstrated that six of ten genes owned 51 prognosis-related methylation sites. Among them, 22 sites played a beneficial role, and 29 played a harmful role (Figure 7A–7F). We performed Kaplan–Meier analysis additionally in order to dialectically indicate the prognostic effect of these methylation sites. Supplementary Figure 5 demonstrates several representative Kaplan–Meier maps; four methylation sites in *BDNF*, two methylation sites in *PIK3R1*, and one methylation site in *TSC2*, *ZNF746*, and *EOMES* could be used to group the patients with HCC in high- and low-risk groups with significant differences in OS (Supplementary Figure 5). The methylation statistics of *BDNF* and *PIK3R1* were significantly correlated with the OS of patients with HCC (Figure 7A, 7D). This was also confirmed by the methylation information from MEX.PRESS website (Figure 7G, 7H).

### Protein-protein interaction network construction and functional enrichment

**Figure 8:**
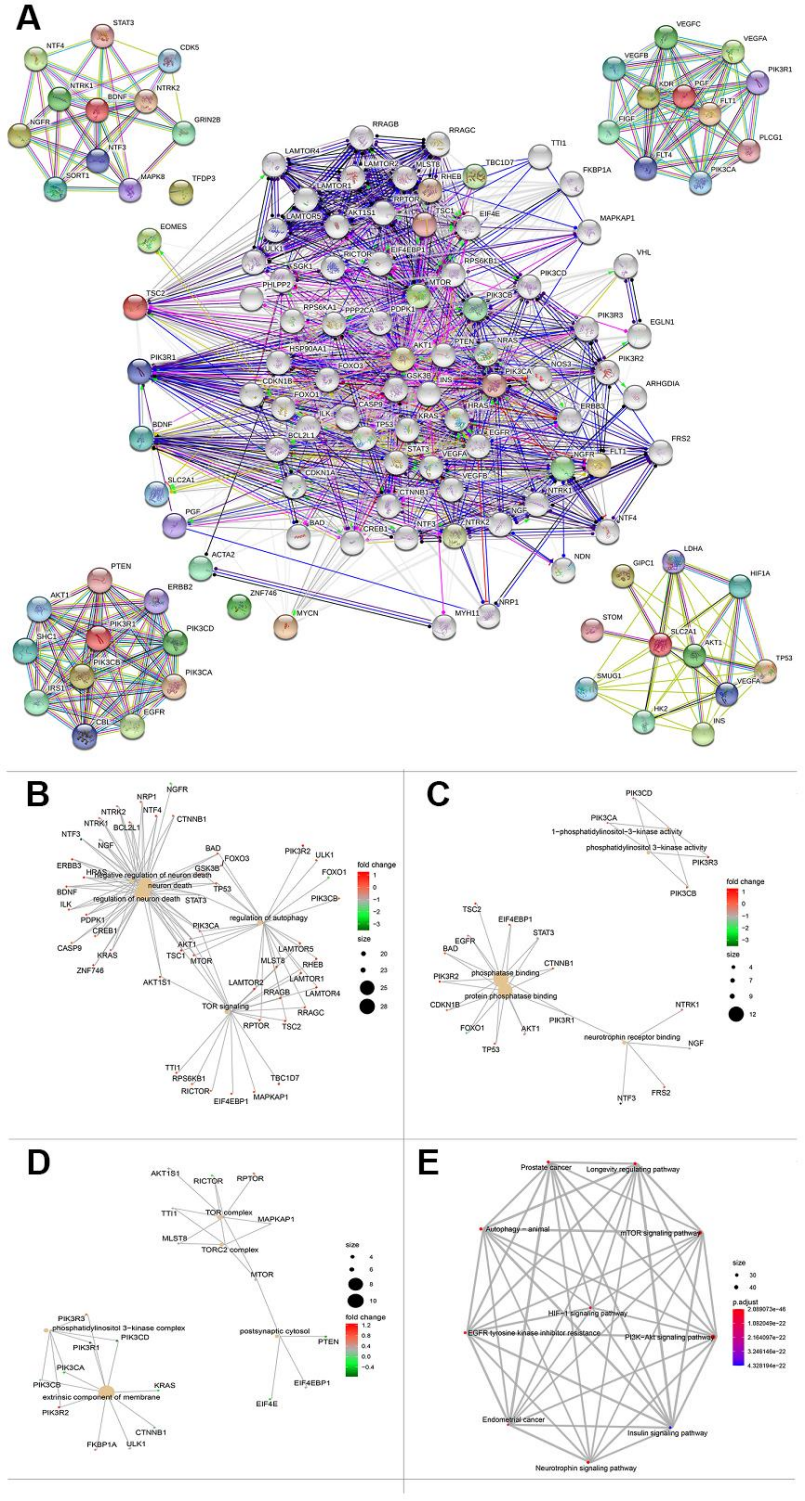
**Protein protein interaction network and functional enrichment of ten gene signature.** (**A**) PPI network of the proteins that have been confirmed to interact directly or indirectly with the 10 risk genes from STRING database with confidence > 0.4 as cutoff. (**B**–**D**) Gene-concept network represents the results for biological process, molecular function and cellular component of Gene ontology analysis based on 80 protein-protein interaction network genes respectively. Each plot shows the enrichment entry with the largest number of genes, and the differences level of each in HCC against normal tissue is represented by the color from green to red. PPI, Protein protein interaction. (**E**) KEGG pathway analysis results for the 80 protein-protein interaction network genes.

To further explore a well-documented protein-protein interaction (PPI) for the signature proteins, we established the protein-protein interaction network using the STRING database. The PPI network added another 70 proteins that had been verified to interact directly or indirectly with the ten genes identified in the current study. The PPI contained 80 nodes and 1,156 edges, and the PPI enrichment *P* value was < 1.0e-16. The top ten genes that interacted with *PGF*, *SCL2AC1*, *PIK3R1*, and *BDNF* are listed in the four corners of the PPI network (Figure 8A). The network also contains some tumor driver genes such as *EGFR*, *VEGFA*, *NGFR*, and *AKT1*. GO functional enrichment results showed that these genes were enriched in molecular functions including 1-PI3K activity and PI3K activity (Figure 8C). The extrinsic membrane component, TOR complex, PI3K complex, TORC2 complex, and postsynaptic cytosol were the top cell component functions enriched (Figure 8D). Additionally, TOR signaling, neuron death, regulation of neuron death, and regulation of autophagy were the top biological processes enriched (Figure 8B). The 3-gene concept network of GO analysis showed that most of the genes in PPI networks were upregulated. KEGG pathway analysis showed that the PPI network of the ten genes was mainly enriched in the PI3K/Akt signaling pathway, mTOR signaling pathway, autophagy-animal pathway, longevity regulating pathway, Epidermal growth factor receptor (EGFR) tyrosine kinase inhibitor resistance pathway, and hypoxia inducible factor (HIF)-1 signaling pathway (Figure 8E).

### Correlation between risk score and immune cell infiltration

**Figure 9:**
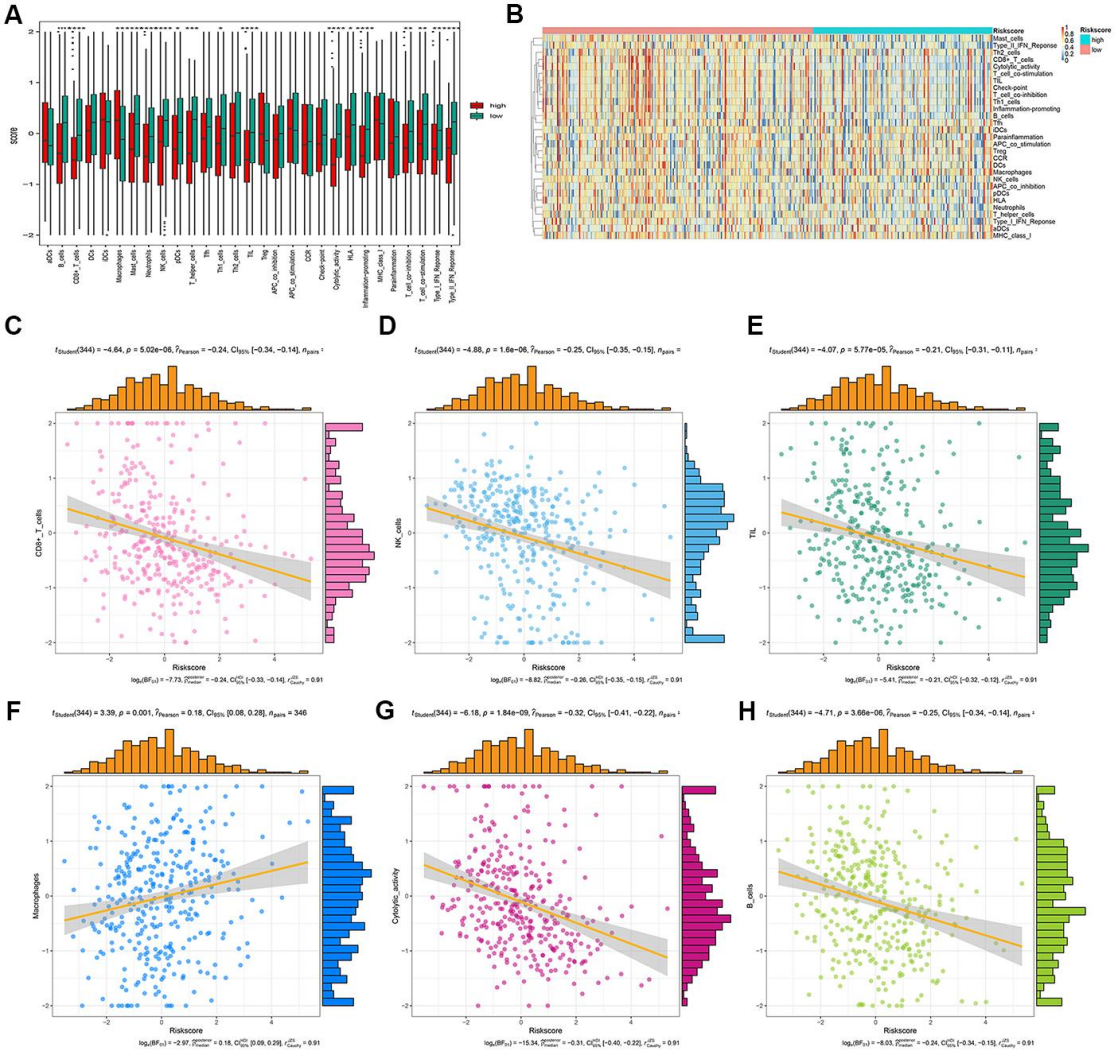
**Correlation analysis between risk score and immune cell infiltration.** (**A**) The number of immune cell infiltration in patients with low-risk and high-risk group. (**B**) Heatmap to visualize the relative abundance of 28 infiltrating immune cell populations in the samples of HCC patients after risk score ordered for low to high. (**C**–**H**) The ggscatterstats plots of the significant correlation between risk score and different immune cells infiltration status.

We analyzed the differences in tumor-infiltrating cells between the high- and low-risk groups. The amount of immune cell infiltration in low-risk patients was significantly higher than that in high-risk patients, and included cell types such as B cells, CD4^+^ T cells, macrophages, Th cells, and tumor-infiltrating lymphocytes (TILs). There were also significant differences in T-cell costimulation signaling and type I IFN signaling between the high- and low-risk groups (Figure 9A). Thus, the risk score can distinguish the type and distribution of immune cell infiltration because significant differences were found in the types of immune cell infiltration between patients with high- and low-risk scores (Figure 9B). The correlation analysis between the gene signature and different types of immune cell infiltration showed that the gene signature was negatively correlated with CD8^+^T cells, B cells, NK cells, TILs, and cytotoxic activity (*P* < 0.001), and positively correlated with macrophages (*P* = 0.001) (Figure 9C–9H).

## DISCUSSION

EMT is a basic process of cell phenotypic transformation and plays an important role tumor metastasis [25]. However, no previous report has examined the application of EMT-related genes to predict the prognosis of HCC. In this study, ten EMT-related genes were identified to predict the prognosis of HCC using bioinformatics analysis. Patients with HCC were divided into high- and low-risk groups according to the risk score, and the prognoses of patients in the high-risk group were found to be significantly worse than those of the low-risk group. Moreover, we found that the 1-, 3-, and 5-year OS of patients with HCC can be effectively predicted by the 10-gene signature. TCGA and ICGC liver cancer cohorts were used to perform internal and external validation. The 10-gene signature was an independent predictive factor and predicted the prognosis better than AJCC stage, tumor grade, and pathologic T, N, and M stages, all of which are commonly used. DCA and ROC curve analysis showed that the risk score of the 10-gene signature was significantly better than that of the clinical indicators. These results indicate that the risk score model has a strong effect in predicting the prognosis of patients with HCC.

Previous studies have reported the prognostic role of different gene signatures for predicting the OS of HCC [11, 26–28]. Ouyang et al. [29] identified a 12-gene signature as a robust marker for HCC OS, and Jiang et al. established a glycolysis-related gene signature that could predict survival in patients with HCC [30]. Although these gene signatures have achieved a positive predictive effect, they did not predict long-term survival, such as 5-year survival for patients with HCC. In the present study, the AUC used this gene signatures were more than 80%, which was better than those of other genetic risk models reported in recent studies [11, 26, 27]. The reason for this difference may be related to the different genes included; indeed, EMT-related genes are more likely to affect the long-term prognosis of patients with HCC. Furthermore, some patients with similar clinical-pathological features have different prognoses, which is likely to be due to the heterogeneity of HCC. The combined application of gene signature and clinical indicators might improve the identification of high-risk patients with HCC. In this study, the AUC for 5-year OS prediction was 0.916 when combining use stages, age, and the 10-gene signature.

Boxplots showed that all ten risk genes were significantly increased. Multivariate regression analysis combing the clinical factors and ten genes showed that all ten genes, except *SLC2A1*, were all independent factors for predicting the prognosis of patients with HCC, while none of the clinical factors showed significant influence (*P* > 0.05). The Kaplan–Meier curves for the high and low-expression groups of the ten genes showed significant differences for all ten genes except *BDNF*. The PPI network contains some tumor driver genes such as *EGFR*, *VEGFA*, *NGFR*, and *AKT1*; these genes are molecular targets of therapy that are commonly used in the clinical setting. Among the top ten enriched pathways in KEGG analysis of the PPI network, the PI3K/Akt signaling pathway, mTOR signaling pathway, and HIF-1 signaling pathway were found to potentially participate in immune cell signaling, suggesting a potential relationship between the 10-gene signature and immunity.

A recent study found that immune cell infiltration was related to EMT and tumor metastasis [31]. Foerster’s study [32] provided the first global characterization of the immune contexture of HCC, and found that it could accurately distinguish between patients with good and poor survival. As a result, potential prognostic immune-related genes were screened, and a novel immune-based prognostic model of HCC was later established by Wang et al. [33, 34]. The 9-gene signature postulated by Wang et al. could reflect the status of the tumor immune microenvironment, and showed significant differences in the types of immune cell infiltration between patients in high- and low-risk groups. Moreover, multiple immune cell infiltration in low-risk patients was significantly higher than that in high-risk patients. The gene signatures were negatively correlated with the infiltration of CD8 ^+^T cells, B cells, NK cells, TILs, and were positively correlated with macrophage infiltration. These results suggested that the gene signature was closely related to immune cell infiltration and affected the prognosis of patients with HCC.

TSC2 is a tumor suppressor, and mutations in TSC2 can lead to tuberous sclerosis complex. When in a complex with TSC1, TSC2 inhibits the growth factor-stimulated phosphorylation of S6K1 and EIF4EBP1 [35]. Caruso et al. reported that inactivating mutations in TSC2 were sensitive to the mTOR inhibitor in liver cancer cell lines [36]. The somatic mutation rate of TSC2 in this study was 3.58%.

ACTA2 is considered a marker of myofibroblasts, and is one of six actin subtypes, including α-smooth muscle actin (SMA), and is involved in smooth muscle contraction. Liao et al.’s study showed that high expression of α-SMA was positively associated with a malignant phenotype and poor prognosis in HCC clinical samples. SLC2A1 (also GLUT1) is a type of glucose transporter that is upregulated in many tumors, and plays an important role in maintaining the growth and reproduction of cancer cells [37]. Sun et al.’s study found that *GLUT1* expression in tumor tissues was significantly higher than that in adjacent non-tumor tissues. Moreover, patients with high expression of *GLUT1* had a poor OS and recurrence-free survival [38].

PGF is a type of cell growth regulator that mainly regulates the response of cells to injury, and maintains the consistency of cell growth, differentiation, and apoptosis. Vandewynckel’s reports showed that the inhibition of PGF exerts antitumor effects and induces vessel normalization [39]. PGF inhibition attenuates PERK activation, likely by tempering hypoxia in HCC via vessel normalization.

MYCN is a member of the Myc family, and its amplification has been reported in numerous tumors. Qin’s study showed that silencing of *MYCN* repressed cell proliferation and induced cell death in HCC cells [40]. Furthermore, a positive correlation was found between *MYCN* expression and recurrence of de novo HCC. Lipid desaturation-mediated ER stress signaling regulates MYCN gene expression in HCC cells.

PIK3R1 is one of the regulatory subunits of PI3K, and plays an important role in cell growth and apoptosis [41]. Ai et al.’s study found that *PIK3R1* was highly expressed in HCC tissues compared with normal tissues [42]; this result was consistent with our analysis from the HPA data. In the current study, both beneficial and harmful methylation sites were found in *PIK3R1*; two methylation sites in *PIK3R1* could predict the OS of HCC patients.

EOMES is an important transcriptional regulator of type I effector T cells, and plays key roles in the regulation of the tumor immune response. Ma et al.’s report showed that PD1^hi^ CD8^+^ T cells were significantly enriched in tumor tissues compared to non-tumor liver tissues. Furthermore, PD1^hi^ CD8^+^ T cells highly expressed transcription factors such as EOMES [43]. BDNF is a brain-derived neurotrophic factor that plays an important role in the growth, differentiation, and regeneration of various neurons. Lam et al.’s study suggested that the BDNF/TrkB system was crucial for tumor angiogenesis and growth, and may be a potential target for antiangiogenic therapy in HCC [44]. The current study found both beneficial and harmful methylation sites in *BDNF*; four methylation sites in *BDNF* could predict the OS of HCC patients. TFDP3 is a new transcription regulator of E2F, which can inhibit the binding and transcriptional activity of E2F with DNA. Jiao et al.’s study indicated that the E2F/TFDP3 complex was involved in cell cycle regulation [45].

ZNF746 is a transcription inhibitor that regulates neuronal death by inhibiting the transcription of peroxisome proliferator-activated receptor gamma coactivator-1 α. Kim’s reports showed that inhibition of ZNF746 can inhibit the invasion and metastasis of non-small cell lung cancer cells [46].

The abovementioned studies show the cancer-related functions of the ten genes identified in the current study, and may provide some clues about the feasibility of these genes as prognostic indicators for HCC. However, the specific mechanism requires further study to verify our findings and the underlying mechanisms.

Two databases were used to validate the genetic risk model, and consistent results were obtained. However, the limitations of this study are as follows: (1) The study relies on the use of a public database and lacks molecular experiments of related genes; (2) the role of the ten genes at the protein expression level in the pathogenesis of HCC requires further investigation; and (3) the accuracy of this gene signature for prediction of prognosis in HCC needs to be verified in future clinical studies.

In conclusion, a prognosis prediction model of an EMT-related gene signature for HCC was established through bioinformatics analysis. This gene signature could effectively predict the OS rate of patients with HCC, and thereby provides a new molecular model for guiding the individualized diagnosis and treatment of HCC.

## Supplementary Material

Supplementary Figures
