# Supplementary material for: Development and validation of epithelial mesenchymal transition-related prognostic model for hepatocellular carcinoma
Source: Aging (Albany NY). 2021 Apr 30;13(10):13822–45. doi: 10.18632/aging.202976 (PMC8202896; doi:10.18632/aging.202976)
Supplement: Supplementary Figures [file aging-13-202976-s001.pdf]

SUPPLEMENTARY FIGURES

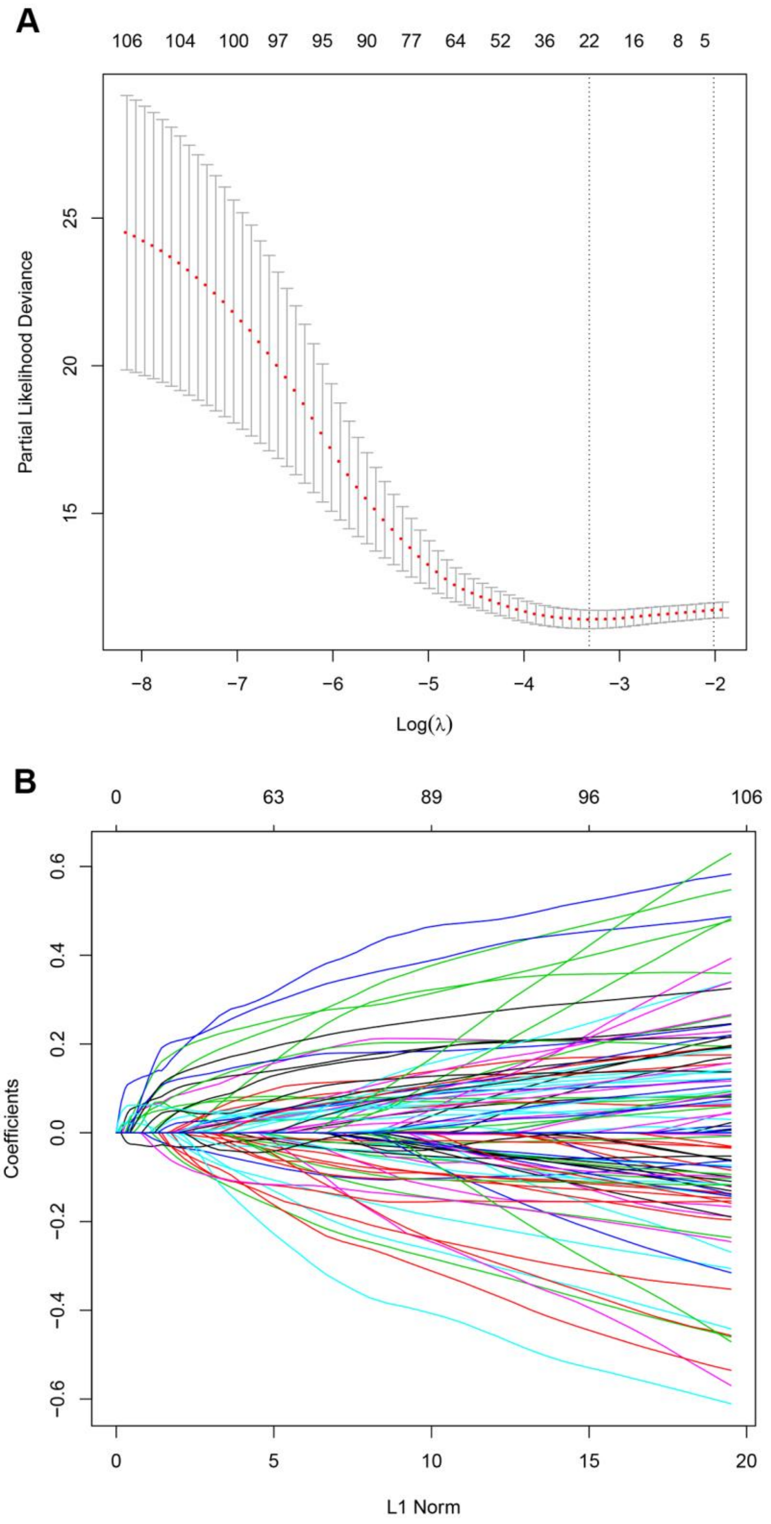

Supplementary Figure 1. Lasso Cox regression analysis of the OS-related genes. Cross-validation plot (**A**) and coefficient against the log-lambda value plot (**B**) for LASSO.

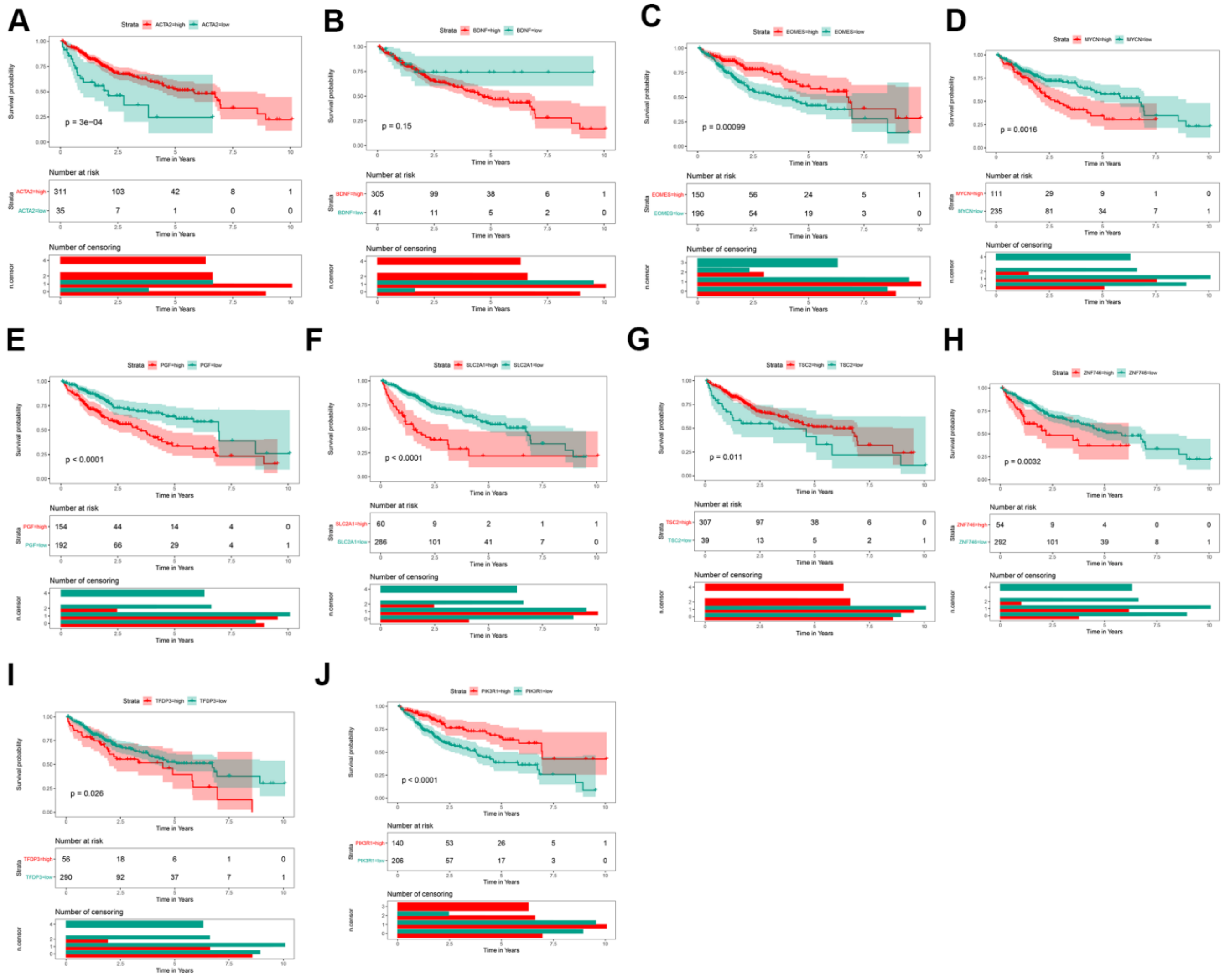

Supplementary Figure 2. Kaplan-Meier survival curve analysis of the 10 genes. (**A**–**J**) Kaplan-Meier survival curve analysis of ACTA2, BDNF, EOMES, MYCN, PGF, SLC2A1, TSC2, ZNF746, TFDP3, and PIK3R1 on HCC patients derived from TCGA LIHC sequencing data respectively.

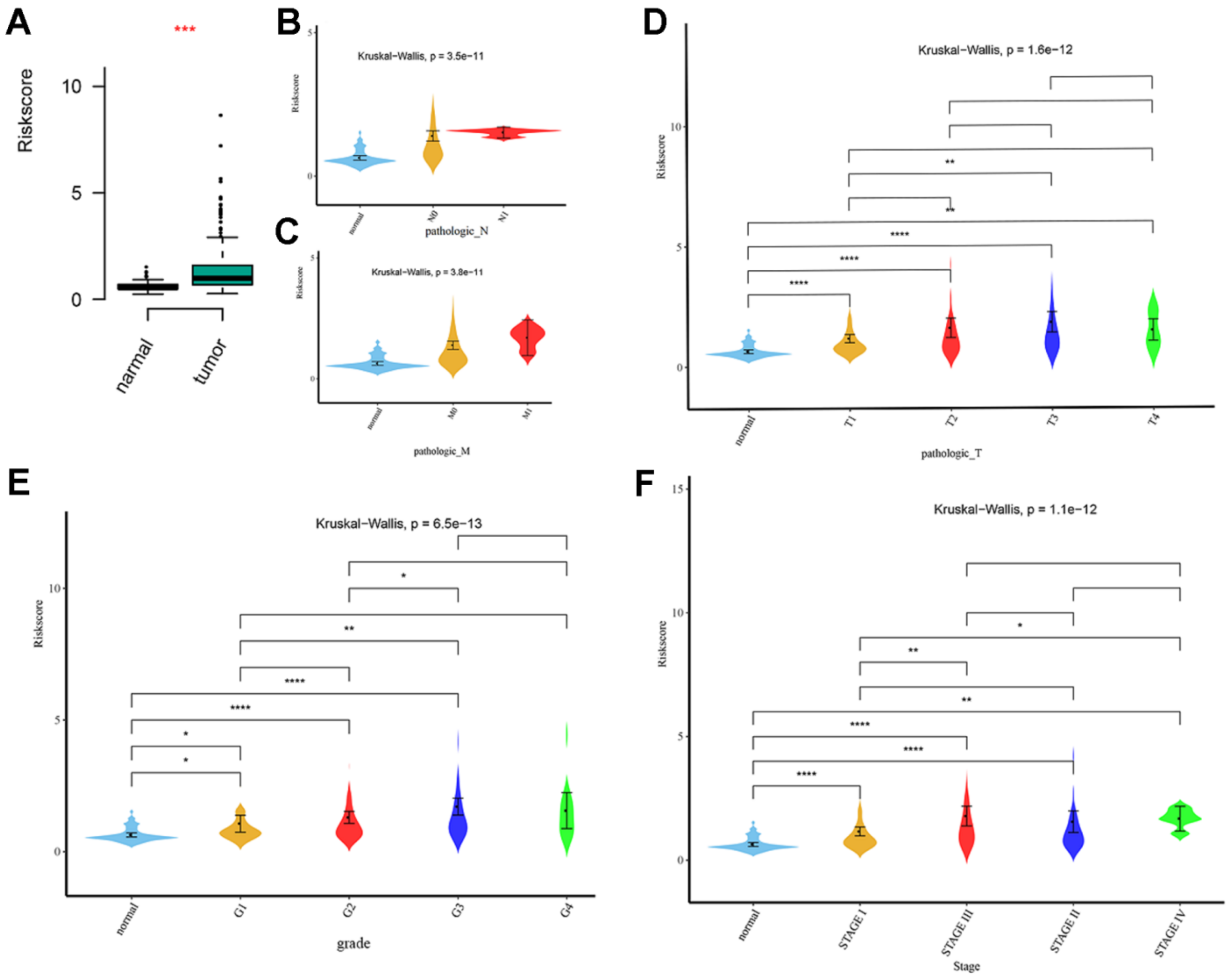

Supplementary Figure 3. The risk score distinguish between normal and tumor tissues, tumor grade and HCC stage. (**A**) Risk score distribution in TCGA-LIHC tumor and normal tissues. (**B**–**F**) Violin plot comparing the levels and distributions of risk score in different Pathologic N, Pathologic M, Pathologic T, tumor grade, and tumor stage.

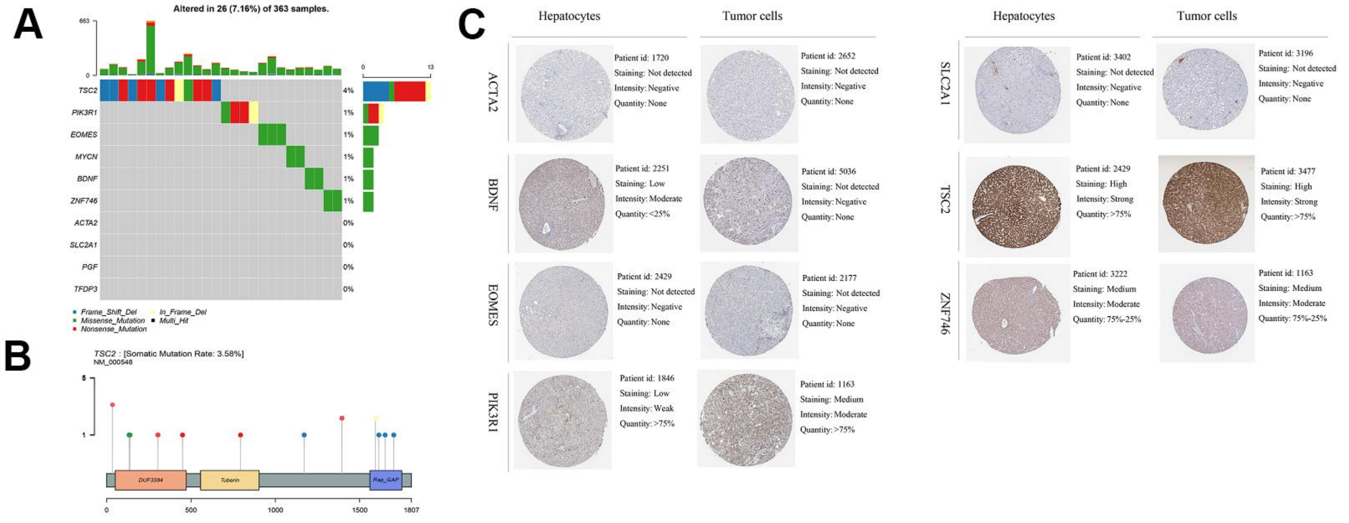

Supplementary Figure 4. Gene mutation analysis of 6 EMT related genes. Gene mutation and protein immunohistochemistry analysis of EMT risk related genes in HCC patients. (**A**) Somatic mutation information of 6 EMT related genes analyzed by "TCGA mutations" package. (**B**) Specific somatic mutation sites distribution of TSC2 in HCC patients analyzed by "TCGA mutations" package. (**C**) Immunohistochemistry staining images of ACTA2, BDNF, EOMES, PIK3R1, SLC2A1, TSC2, ZNF746 in HCC tumor tissues and normal tissues from the Human Protein Atlas database.

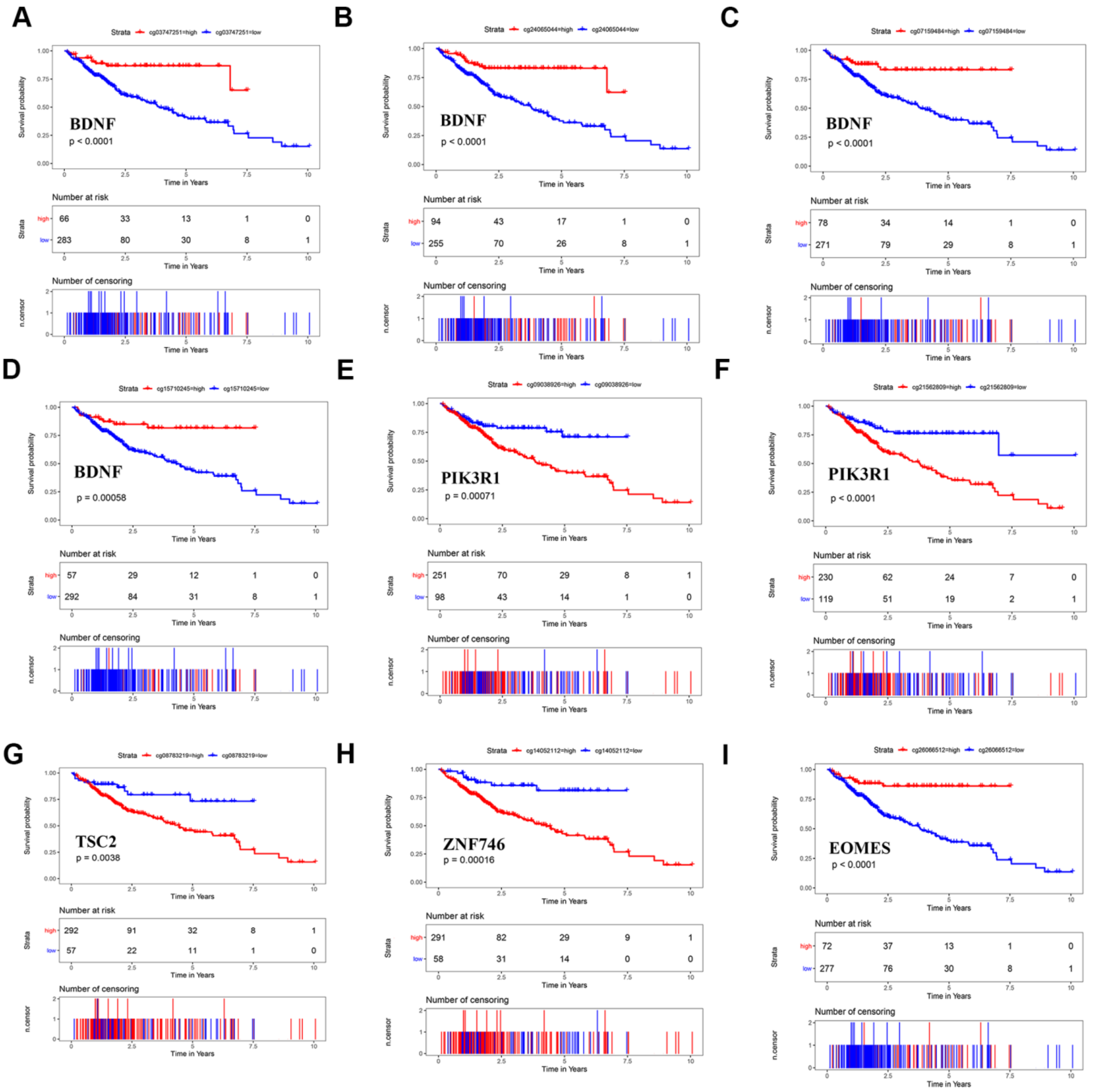

Supplementary Figure 5. KM survival analysis of methylation sites in BDNF, PIK3R1, TSC2, ZNF746 and EOMES gene. (**A**–**D**) Kaplan-Meier survival curve analysis of four significant OS related BDNF methylation sites derived from TCGA-LIHC DNA methylation data respectively. (**E**, **F**) Kaplan-Meier survival curve analysis of two significant OS related PIK3R1, methylation sites derived from TCGA-LIHC DNA methylation data respectively. (**G**) Kaplan-Meier survival curve analysis of one significant OS related TSC2 methylation sites derived from TCGA-LIHC DNA methylation data. (**H**) Kaplan-Meier survival curve analysis of one significant OS related ZNF746 methylation sites derived from TCGA-LIHC DNA methylation data. (**I**) Kaplan-Meier survival curve analysis of one significant OS related EOMES methylation sites derived from TCGA-LIHC DNA methylation data.
